# Case Commentary: When one target is not enough—PBP3 insertions and target redundancy in *Escherichia coli*

**DOI:** 10.1128/aac.00053-26

**Published:** 2026-04-06

**Authors:** Madison E. Stellfox, Yohei Doi

**Affiliations:** 1 Division of Infectious Diseases, University of Pittsburgh School of Medicine 12317 , Pittsburgh, Pennsylvania, USA; 2 Departments of Microbiology and Infectious Diseases, Fujita Health University School of Medicine 89305, Aichi, Japan; Houston Methodist Hospital and Weill Cornell Medical College, Houston, Texas, USA

**Keywords:** PBP insertions, target redundancy, beta-lactam resistance

## Abstract

This challenging clinical case highlights the impact of PBP3 insertions in *Escherichia coli*, which reduce susceptibility to key β-lactam agents and complicate treatment, particularly in the setting of NDM production (C. Fabrizio, F. Valzano, S. Giuliano, E. Morelli, et al., Antimicrob Agents Chemother 70:e00887-25, 2026, https://doi.org/10.1128/aac.00887-25). Clinical improvement was achieved with imipenem–relebactam plus aztreonam, supporting the idea that targeting multiple penicillin-binding proteins can overcome functional redundancy. These findings emphasize the clinical relevance of PBP3-mediated resistance and the need for treatment strategies that address complex β-lactam resistance in Gram-negative pathogens.

## COMMENTARY

Penicillin-binding proteins (PBPs) build and maintain the bacterial cell wall, and β-lactams exert antibacterial activity by inhibiting PBPs, disrupting cell wall architecture and causing bacterial death (1). Historically, β-lactam resistance in Gram-negative pathogens has been attributed to mechanisms that reduce the effective periplasmic concentration of the drug, such as β-lactamase production, decreased membrane permeability, and increased efflux. Implicit in this framework is the assumption that PBP targets remain structurally stable and therapeutically reliable. However, accumulating genomic and phenotypic data in *Escherichia coli* now challenge this premise. Four-amino-acid insertions in PBP3, most commonly YRIN or YRIK between residues 333 and 334 of the β2b–β2c loop adjacent to the transpeptidase active site, are associated with reduced susceptibility to multiple PBP3-targeting β-lactams, including aztreonam, cefepime, and ceftazidime (2–5).

First reported in 2015 in association with reduced aztreonam–avibactam susceptibility, these insertions are increasingly identified in globally disseminated, high-risk *E. coli* lineages such as ST167, ST405, ST410, and ST648 (4, 6). These lineages frequently co-produce NDM-group metallo-β-lactamases along with CMY-group cephalosporinases or CTX-M-group extended-spectrum β-lactamases (ESBLs) (6). The convergence of target modification and potent β-lactamase production can compromise recommended regimens for metallo-β-lactamase-producing Enterobacterales, including ceftazidime–avibactam plus aztreonam and, to a lesser extent, cefiderocol (3, 7–10).

Fabrizio and colleagues illustrate this clinical challenge in a patient with peritonitis caused by a carbapenem-resistant *E. coli* isolate harboring a PBP3 insertion (11). After its initial identification, the patient was placed on ceftazidime-avibactam and aztreonam but failed to improve. Using expedited synergy testing by Etest, subsequently confirmed by checkerboard assays, the authors observed additive activity with imipenem–relebactam plus aztreonam. In contrast, no such additivity was observed with the ceftazidime–avibactam plus aztreonam combination. Accordingly, therapy was changed to imipenem–relebactam plus aztreonam (with adjunctive tigecycline), leading to clinical improvement. Whole-genome sequencing identified the isolate as ST167 carrying the YRIN insertion in PBP3. Molecular dynamics simulations suggested that this insertion constricts the region near the transpeptidase active site, potentially limiting β-lactam access (4, 11). The isolate also produced NDM-5 and CMY-184, which likely further diminished the activity of ceftazidime–avibactam plus aztreonam.

PBPs comprise a diverse family of enzymes classified by molecular weight and their role in peptidoglycan synthesis, cross-linking, and maintenance. While they perform distinct functions, they also provide functional redundancy essential for cell wall integrity (1). In *E. coli*, PBP3 is enriched at the site of cell division and is required for septation, whereas PBP2 governs lateral wall elongation and maintenance of bacillary shape (12). Many widely used β-lactams derive activity predominantly from inhibition of PBP3, rendering them vulnerable to structural target alterations. Carbapenems, in contrast, exhibit broader PBP binding profiles (2, 13, 14). Relevant to this case, imipenem binds multiple essential PBPs in *E. coli*, particularly PBP2, but also PBP1a, PBP1b, and PBP3, thereby distributing antibacterial activity across several targets rather than relying on a single PBP (15). Assuming comparable β-lactamase inhibition by relebactam and avibactam, this broader target engagement may explain the retained activity of imipenem–relebactam plus aztreonam against selected NDM-producing *E. coli* with PBP3 insertions by exploiting intrinsic target redundancy.

Important questions remain. Additive activity was not observed with meropenem–vaborbactam plus aztreonam, suggesting that not all carbapenem–β-lactamase inhibitor combinations behave similarly. This discrepancy may reflect differences in the PBP binding profile or membrane permeability between meropenem and imipenem, the level of β-lactamase inhibition by relebactam versus vaborbactam (particularly of the uncharacterized CMY-184), or the contribution of additional mechanisms. Moreover, the expanding spectrum of PBP3 insertion variants beyond YRIN and YRIK raises questions about the generalizability of this strategy across the broader global population of PBP3-mutant *E. coli* (6).

Notably, this specific pattern of PBP3 insertions has thus far been confined to *E. coli,* suggesting species-specific structural or evolutionary constraints. Nevertheless, PBP alterations are increasingly recognized as contributors to β-lactam resistance across other Gram-negative pathogens. Substitutions in PBP3 compromise sulbactam activity in *Acinetobacter baumannii* (16) and reduce susceptibility to multiple β-lactams in *Pseudomonas aeruginosa* (17). Combinatorial substitutions in PBP2 have been linked to carbapenem resistance (18) and reduced direct PBP2 engagement by newer diazabicyclooctane (DBO) β-lactamase inhibitors in *E. coli* (19). Collectively, these examples illustrate that PBPs are dynamic determinants of β-lactam activity rather than static drug targets in Gram-negative organisms.

The emergence of PBP-mediated resistance in multidrug-resistant Gram-negative pathogens underscores the limits of strategies focused heavily on β-lactamase inhibition. As enzyme-mediated resistance and target modification converge, optimizing inhibitor chemistry alone may be insufficient. Instead, therapeutic strategies that account for PBP engagement profiles and exploit target redundancy may better preserve the clinical utility of the β-lactam class. In addition to the case report highlighted above (11), *in vitro* studies in *A. baumannii* and *Klebsiella pneumoniae* demonstrate that combining β-lactams and β-lactamase inhibitors with complementary PBP binding profiles enhances bactericidal activity even against carbapenem-producing isolates (20, 21). This indicates that even significant resistance may be surmountable with rationally designed combinations that maximize drug-target engagement and overwhelm complex resistance mechanisms.

At the same time, this approach presents a critical therapeutic and stewardship dilemma. If we continue to rely on β-lactam agents with a single dominant target, then selection for structural modifications of that target, such as these PBP3 insertions, can undermine our best β-lactam–β-lactamase inhibitor regimens. Regimens that distribute activity against multiple essential PBPs may buffer against single-target escape, but at the cost of intensifying selection for carbapenemase-mediated resistance. Thus, exploiting target redundancy must remain a deliberate, genotype-informed strategy.

In an era of escalating antibiotic resistance, exploiting PBP target redundancy should be viewed as a precision medicine tactic rather than an empiric escalation pathway. Mechanism-guided selection of combinations such as imipenem–relebactam plus aztreonam may be justified in well-characterized cases, but routine escalation to carbapenem-based combinations would be counterproductive. These tensions highlight the need for improved molecular diagnostics and expanded access to whole-genome sequencing to rapidly identify uncommon resistance genotypes, including PBP3 insertions, and guide rational combination therapy. Leveraged judiciously, target redundancy may extend, rather than erode, the lifespan of existing β-lactam therapies.
